# Emergent gamma synchrony in all-to-all interneuronal networks

**DOI:** 10.3389/fncom.2015.00127

**Published:** 2015-10-13

**Authors:** Shivakeshavan Ratnadurai-Giridharan, Pramod P. Khargonekar, Sachin S. Talathi

**Affiliations:** ^1^J. Crayton Pruitt Family Department of Biomedical Engineering, University of FloridaGainesville, FL, USA; ^2^Electrical and Computer Engineering, University of FloridaGainesville, FL, USA; ^3^Qualcomm ResearchSan Diego, CA, USA

**Keywords:** gamma, STDP, computational, interneuron, plasticity, network synchronization

## Abstract

We investigate the emergence of in-phase synchronization in a heterogeneous network of coupled inhibitory interneurons in the presence of spike timing dependent plasticity (STDP). Using a simple network of two mutually coupled interneurons (2-MCI), we first study the effects of STDP on in-phase synchronization. We demonstrate that, with STDP, the 2-MCI network can evolve to either a state of stable 1:1 in-phase synchronization or exhibit multiple regimes of higher order synchronization states. We show that the emergence of synchronization induces a structural asymmetry in the 2-MCI network such that the synapses onto the high frequency firing neurons are potentiated, while those onto the low frequency firing neurons are de-potentiated, resulting in the directed flow of information from low frequency firing neurons to high frequency firing neurons. Finally, we demonstrate that the principal findings from our analysis of the 2-MCI network contribute to the emergence of robust synchronization in the Wang-Buzsaki network (Wang and Buzsáki, 1996) of all-to-all coupled inhibitory interneurons (100-MCI) for a significantly larger range of heterogeneity in the intrinsic firing rate of the neurons in the network. We conclude that STDP of inhibitory synapses provide a viable mechanism for robust neural synchronization.

## 1. Introduction

Cortical gamma rhythms (30–80 Hz) are correlated with diverse brain functions such as memory formation (Singer and Gray, 1995), linguistic processing (Pulvermller et al., 1995), and associative learning (Ritz and Sejnowski, 1997; Miltner et al., 1999). Two commonly known mechanisms for gamma oscillations are Pyramidal-Interneuronal Network Gamma (PING) and Interneuron Network Gamma (ING).

Interneurons play critical roles for gamma rhythm generation in both mechanisms. However, in ING, interneurons are solely responsible for gamma frequency activity (Bartos et al., 2002). Additionally, there is evidence that interneurons show phase-locked activity with gamma oscillations (Bartos et al., 2007). In particular, perisomatic inhibition caused by fast-spiking parvalbumin (PV) immunoreactive basket cell interneurons (Lytton and Sejnowski, 1991) have been implicated in the generation of cortical and subcortical gamma oscillations (Bartos et al., 2007).

Previous theoretical works have shown that synchronization is possible in interneuronal networks (Wang and Rinzel, 1992; van Vreeswijk et al., 1994), however, this synchronization is not resilient to even slight heterogeneity or noise in the network (Wang and Buzsáki, 1996). In biological systems, inherent noise and heterogeneities in neuronal firing rates are likely to be present. Investigations by Kopell and Ermentrout (2004) have shown that gap junctions are one way by which interneuronal network synchronization can be robust against heterogeneities and noise in the network.

Evidence from literature suggests a strong correlation between Hebbian learning and Gamma rhythms (Miltner et al., 1999). Indeed, studies have established a link between glutamatergic synaptic plasticity and gamma oscillations (Whittington et al., 1997; Traub et al., 1998). More recently, synaptic plasticity of inhibitory Gamma-Aminobutyric acid (GABA) synapses was observed and characterized (Haas et al., 2006). This led us to question whether inhibitory synaptic plasticity could also play a role in generation of gamma oscillations.

In our earlier works, we demonstrated that inhibitory plastic synapses could improve in-phase synchronization in the presence of firing rate heterogeneity in a simple network of uni-directionally coupled interneurons (UCI) (Talathi et al., 2008). In the current paper, we investigate how the presence of synaptic plasticity can increase the range of firing rate heterogeneity for which mutually-coupled parvalbumin positive interneuronal (MCI) networks can synchronize at gamma frequencies. We begin with analyzing in-phase synchrony of a two interneuron MCI network (2-MCI) and prove that the analysis holds even for a much larger network (100-MCI). We demonstrate that the spike time response curve (STRC) of parvalbumin positive interneurons exhibit a strong second order component. We present a novel approach to account for the second order STRC component in the analysis of 1:1 in-phase synchronization of the 2-MCI parvalbumin positive interneuronal network.

The organization of the paper is as follows: the Methods Section presents the mathematical model for the network, the interneuron, and the synapse used in our studies. We analyze two types of heterogeneity in the interneuronal networks: (1) temporal heterogeneity; which corresponds to the heterogeneity in the intrinsic firing rates of the coupled interneurons, and (2) structural heterogeneity; which corresponds to the heterogeneity in the synaptic coupling strength. We introduce a few network measures in order to quantify the network state in terms of its connectivity and/or synaptic strengths. We then introduce a novel method for using STRC's with a strong second order component to estimate the spike times for a periodically firing neuron that receives periodic synaptic inputs. Finally, we introduce the empirical STDP rule (Haas et al., 2006; Talathi et al., 2008) used in our investigations of the emergence of synchronization in MCI networks.

In the Results Section, we systematically analyze the synchronization domain of a 2-MCI system in terms of temporal and structural heterogeneity. We show how heterogeneity and initial values of synaptic strengths determine the final synchronization state of the 2-MCI system. The 1:1 domain of synchronization identified by this analysis allows us to determine how STDP must evolve synaptic strengths in order to achieve 1:1 synchronization. We then use the STDP rule and STRC of the interneuron model to derive a nonlinear map for the evolution of the 2-MCI system to a state of in-phase synchronization. Finally, we demonstrate that the principal findings from our analysis of the 2-MCI network hold for larger MCI networks.

## 2. Materials and methods

### 2.1. The network model

Each neuron in the network of all-to-all coupled inhibitory interneurons is modeled based on a single compartment model for parvalbumin positive inhibitory neuron developed by Wang and Buzsáki (1996) with a fast sodium channel, a delayed rectifier potassium channel and a leak channel. The dynamical equation for the model neuron is given by,

(1) $$\begin{array}{l} C\frac{dV_{j}(t)}{dt}=I_{j}^{DC}+g_{Na}m_{\infty }^{3}h(t)(E_{Na}-V_{j}(t))\\ \;+g_{K}n^{4}(t)(E_{K}-V_{j}(t))+g_{L}(E_{L}-V_{j}(t))\\ \;+\sum_{i}a_{ij}g_{ij}(t)s_{i}(t)(E_{I}-V_{j}(t)) \end{array}$$

where *C* = 1 μ F/cm^2^. *V*_*j*_(*t*) is the membrane potential of the jth neuron (*j* = {1, 2, ⋯*N*}). The external current, $I_{j}^{DC}$ is given as follows:

(2) $$I_{j}^{DC}=I_{ref}+\left(j-\frac{N+1}{2}\right)\left(\frac{H\times I_{ref}/100}{N-1}\right)$$

where, the parameter *H* controls the degree of heterogeneity in the intrinsic firing activity of the interneurons in the network. When *H* > 0, $I_{j}^{DC}<I_{j+1}^{DC}$. Since neuron *j*'s firing period is determined by $I_{j}^{DC}$, this means that neuron *j* fires slower than neuron *j* + 1. The actual value of H determines the amount by which neighboring neurons differ in their *I*^*DC*^-values (and correspondingly, firing periods), as given in Equation (2). Hence for a larger *H*, there is a larger range of firing periods across the neurons in the network, corresponding to a larger temporal heterogeneity. The parameter *I*_*ref*_ = 1 μA/cm^2^ in Equation (2) is set such that the mean intrinsic frequency of firing for the neurons in the network is ≈ 60 Hz (Wang and Buzsáki, 1996). *E*_*r*_ (*r* = Na, K, L) are reversal potentials of the sodium and potassium ion channels and the leak channel, respectively. *E*_*I*_, is the reversal potential of the fast GABAergic inhibitory synapse. *g*_*r*_ (*r* = Na, K, L) represent the conductance of sodium, potassium, and the leak channel, respectively. The steady state activation for sodium current, *m*_∞_ = α_*m*_∕(α_*m*_ + β_*m*_). The inactivation variable for sodium channel *h*(*t*) and the activation variable for potassium current *n*(*t*) satisfy the following first order kinetic equation: $\frac{dX\left(t\right)}{dt}=\phi \left(\alpha_{X}\left(V_{j}\left(t\right)\right)\left(1-X\left(t\right)\right)-\beta_{X}\left(V_{j}\left(t\right)\right)X\left(t\right)\right)$, where *X*(*t*) = *h*(*t*), *n*(*t*) with ϕ = 5. The functions α_*X*_ and β_*X*_ are given by:

(3) $$\begin{array}{l} \begin{array}{cc} \alpha_{m}(x)=\frac{-0.1(x+35)}{e^{-0.1(x+35)}-1} & \beta_{m}(x)=4e^{-(x+60)/18} \end{array}\\ \begin{array}{cc} \alpha_{h}(x)=0.07e^{-(x+58)/20} & \beta_{h}(x)=\frac{1}{e^{-0.1(x+28)}+1} \end{array}\\ \begin{array}{cc} \alpha_{n}(x)=\frac{-0.01(x+34)}{e^{-0.1(x+34)}-1} & \beta_{n}(x)=\frac{0.125}{e^{-(x+44)/88}} \end{array} \end{array}$$

The strength of inhibitory synaptic coupling from the pre-synaptic neuron *i* onto the post-synaptic target neuron is modeled via the variable *g*_*ij*_(*t*). In the presence of spike timing dependent plasticity; the coupling strength evolves depending on the plasticity rule as a function of the relative timing of spiking activity between the neurons *i* and *j*. In absence of synaptic plasticity the synaptic coupling strength is set as:

(4) $$g_{ij}=\frac{g_{0}}{N}\left(1+\frac{\eta }{100}\text{sgn}(i-j)\right)$$

where |η| ≤ 100. For all the results presented unless otherwise stated, *g*_0_ = 0.1 mS/cm^2^. The parameter η regulates the structural heterogeneity in the network such that for η ≠ 0, there is asymmetry in the synaptic coupling strengths of the two mutually coupled neurons. By design, the asymmetry is such that for η > 0, the strength of synapse from neuron with lower index is smaller than that of the synapse from neuron with higher index label. The variable *a*_*ij*_ defines the topology of the inhibitory neuronal network such that, for an all-to-all coupled network, we have *a*_*ij*_ = 1 ∀*i* ≠ *j* and *a*_*ij*_ = 0 otherwise for all *i, j* = {1, 2, ⋯*N*}. The variable *s*_*i*_(*t*) models the fraction of bound neurotransmitters onto the post-synaptic neuron GABAergic receptors resulting from a spike elicited by the pre-synaptic neuron i, and satisfies the following first order kinetic equation, Abarbanel et al. (2003) and Talathi et al. (2008),

(5) $${\dot{s}}_{i}(t)=\frac{S_{0}(V_{i}(t))-s_{i}(t)}{\hat{\tau }(S_{I}-S_{0}(V_{i}(t))}$$

The kinetic equation for *s*_*i*_(*t*) involves two time constants, $\tau_{R}=\hat{\tau }\left(S_{I}-1\right)=0.1$ ms, the docking time for the neurotransmitter binding and $\tau_{D}=\hat{\tau }S_{I}=10$ ms, the undocking time constant for the neurotransmitter binding. Finally, *S*_0_(*x*) is the sigmoidal function given by, *S*_0_(*x*) = 0.5(1 + tanh(120(*x* − 0.1))).

In order to quantify the influence of synaptic plasticity on the structure of synaptic connectivity in the network, we define the following network measures:

(a) Link imbalance: The difference in synaptic strengths of the two coupled neurons,

(6) $$L_{ij}=-L_{ji}=a_{ij}g_{ij}-a_{ji}g_{ji}$$

(b) Neuronal strength: The sum of synaptic strength of all outgoing synapses from the neuron *i*,

(7) $$G_{i}=\sum_{j}a_{ij}g_{ij}$$

For all the networks considered here, the neurons in the network are labeled in ascending order of input DC current such that label 0 is assigned to the neuron with lowest intrinsic firing rate while label *N* − 1 is assigned to neuron with the highest intrinsic firing rate.

### 2.2. Spike time response curve

As a measure of the influence of synaptic input on the firing times of a given neuron *i*, we define the spike time response curves (STRC's) $\Phi_{ij}\left(\tau_{R},\tau_{D},E_{R},T_{i0},g,\delta t\right)=\frac{T_{ij}-T_{i0}}{T_{i0}}$ (Acker et al., 2004; Oprisan et al., 2004; Talathi et al., 2010), where *T*_*i*0_ is the intrinsic period of spiking, and *T*_*ij*_ represents the length of the jth spiking cycle from the cycle *j* = 1 in which the neuron receives synaptic input at time 0 < δ*t* <*T*_*i*0_. A synaptic perturbation can either advance or delay the occurrence of an impending spike depending on the bifurcation character of the neuron (Ermentrout, 1996) and the type of synaptic perturbation (excitatory vs. inhibitory). The number of firing cycles *j*, for which the effect of synaptic perturbation lasts, depends on the synaptic parameters: the synaptic rise time τ_*R*_, the synaptic decay time τ_*D*_, the reversal potential of the synapse *E*_*R*_, and the synaptic conductance *g*. For fast hyperpolarizing inhibitory synapse (*E*_*R*_ = −75 mV), as considered in this study, a synaptic input delays the time of occurrence of subsequent spike such that Φ_*i*1_ ≥ 0 ∀δ*t* > 0, Φ_*i*2_ > 0 for δ*t* → *T*_*i*0_, and Φ_*ij*_ = 0 ∀*j* > 2 (Talathi et al., 2010). We note that in all further calculations, unless otherwise mentioned, we suppress the dependence of Φ on τ_*R*_, τ_*D*_, *E*_*R*_, and the intrinsic firing period of neuron *T*_0_ and define Φ(τ_*R*_, τ_*D*_, *E*_*R*_, *T*_0_, *g*, δ*t*) ≡ Φ(*g*, δ*t*).

**Figure 1:**
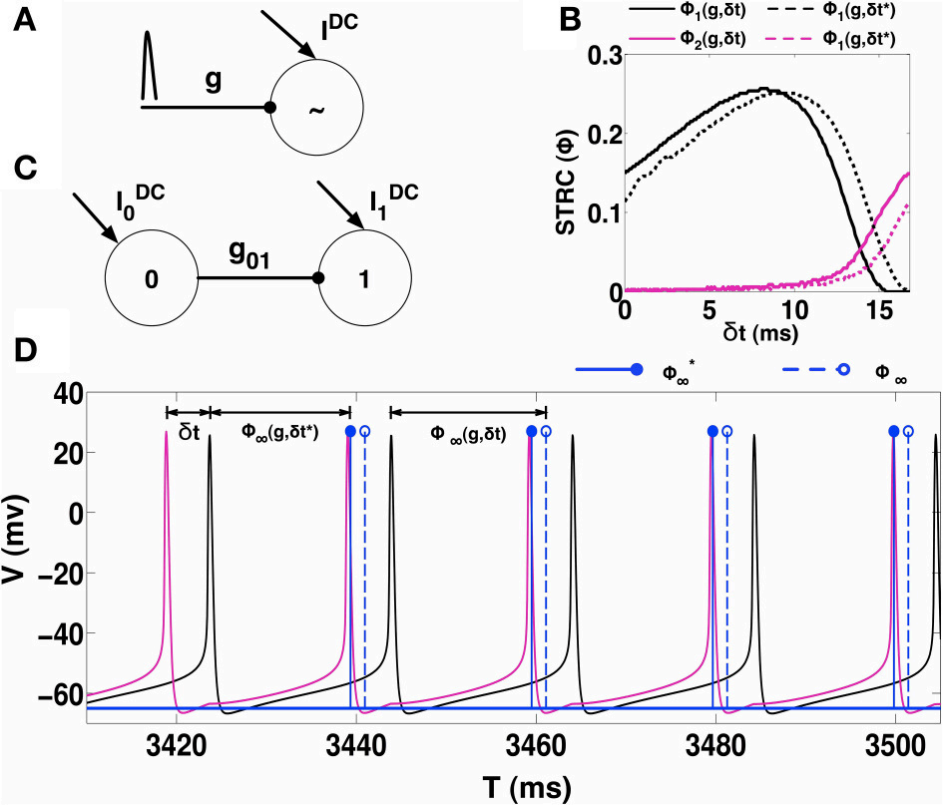
**STRC Calculations. (A)** Schematic for determining the STRC using the standard method. **(B)** The neuron firing intrinsically at 60 Hz, receives inhibitory synaptic perturbation with synaptic parameters: *E*_*R*_ = −75 mV, τ_*R*_ = 0.1 ms, τ_*D*_ = 5 ms, and *g* = 0.1 mS/cm^2^. The original STRC is shown in solid colors while the STRC with the included phase correction term (δt^*^) is represented by the dotted lines. **(C)** Schematic of an uni-directionally coupled two-interneuron network. **(D)** For *H* = 30 the neurons exhibit phase-locked 1:1 synchronization. The spike from Neuron 0 (black voltage trace) synaptically perturbs neuron 1 (magenta voltage trace) at a fixed δ_*t*_ during every firing cycle of neuron 1. The solid blue lines indicate the spike-time shift calculated using the STRC that incorporates the correction factor. The dotted blue lines indicate the spike-time shift calculated using the original STRC's without any correction term.

We begin by estimating STRC's using the direct method as follows: consider neuron *i*, firing regularly with period *T*_*i*0_. The neuron is perturbed through an inhibitory synapse at time δ*t* after the neuron has fired a spike at reference time zero (see Figure 1A). The spiking time for a neuron is considered to be the time when its membrane voltage V, crosses a threshold (set to 0 mV in all the calculations presented here). As a result of this perturbation, the neuron fires the next spike at time *t*_1_, representing the first cycle after perturbation of length *T*_*i*1_ ≠ *T*_*i*0_. Depending on the properties of the synapse, i.e., *g*_*s*_, τ_*R*_, τ_*D*_, and *E*_*R*_; the length of subsequent cycles might change. In Figure 1B, we plot the first two STRC's Φ_*i*1_ (solid black line) and Φ_*i*2_ (solid magenta line), estimated by using the procedure described above for δ*t* ∈ [0, *T*_*i*0_). We note the presence of a non-zero second order STRC component for the choice of the parameters of the neuron.

In the direct method for computing STRC's, we use a single perturbation to observe the changes in spike times of a periodically firing neuron. These changes in spike times can be used to predict future spike times. If Φ_*i*2_ ≈ 0, then a single perturbation arriving at δ*t*, following the last spike at reference time *t*_0_, can be assumed to produce a spike time shift such that the next spike occurs at time *t*_1_ = *t*_0_ + *T*_*i*0_(1 + Φ_*i*∞_(*g*, δ*t*)), where Φ_*i*∞_(*g*, δ*t*) = Φ_*i*1_(*g*, δ*t*) + Φ_*i*2_(*g*, δ*t*) (Oprisan and Canavier, 2001). When a significant higher order STRC component (Φ_*i*2_) is present, Φ_*i*∞_(*g*, δ*t*) can no longer be expressed as a linear sum of the first and second order STRC components (Talathi et al., 2010). With a significant second order STRC component, the neuron requires at least two firing cycles before it resets to its original firing period. In such conditions, the assumption of linear second order STRC correction to the estimated future spike time is no longer valid (Talathi et al., 2010).

We demonstrate this issue using a 2-UCI network as shown in Figure 1C. Here, neuron 1, which fires periodically, is also periodically perturbed by neuron 0 via an inhibitory synapse. We consider a specific case of the 2-UCI network where firing rate heterogeneity *H* = 30 (Equation 2) and phase locked 1:1 synchrony exists. The voltage traces of neuron 0 and 1 are shown as black and magenta solid curves, respectively. We see in Figure 1D that when the shift in spike times are predicted using *T*_*i*1_ = *T*_*i*0_(1 + Φ_*i*∞_(*g*, δ*t*)), there is a noticeable error between predicted spike times (blue dotted stems) and the actual spike times (black solid curve). We next sought to empirically quantify the error between predicted and actual spike times as a function of δ*t*.

Previously, Talathi et al. (2008) derived a STRC based theoretical map for when a 2-UCI network (see Figure 1C) exhibits steady state 1:1 phase locked synchrony. The map is expressed as:

(8) $$T_{00}-T_{10}=T_{10}\Phi_{1\infty }(g_{01},\delta t_{s})$$

where, *T*_00_ and *T*_10_ represent the firing periods of neurons 0 and 1, respectively, Φ_1∞_(*g*_01_, δ*t*_*s*_) is the STRC calculated for the steady-state perturbation time δ*t*_*s*_, which is effectively the spike time difference between the two neurons. The spike time difference could also be measured directly by observing simulation results of the 2-UCI network in a state of 1:1 synchrony, which we denote as δt^*^. When the STRC's second order component is weak, the theoretical predictions match closely with the simulations and we get $\delta t^{*}\approx \delta t_{s}$. However, when the STRC's second order component is strong, there is a noticeable deviation between the map's δ*t*_*s*_ and simulation's δt^*^. We term this error as $\bar{\delta t}=\delta t^{*}-\delta t_{s}$.

**Figure 2:**
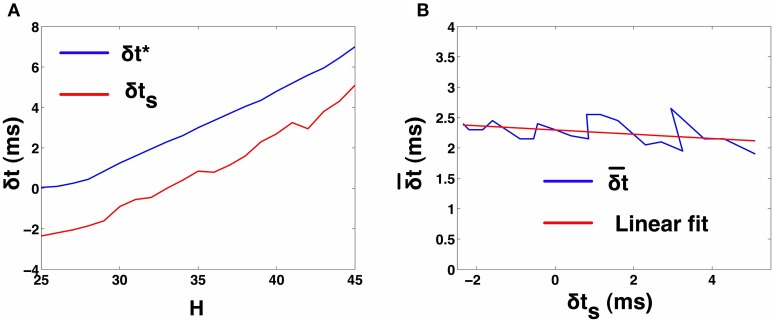
**2-UCI Simulation and STRC Based Spike Time Calculations. (A)** Shows the variation of simulation spike time difference δt^*^ and STRC based map predicted δ*t*_*s*_ as *H* is varied. The negative values for δ*t*_*s*_ is due to the inaccuracy of the standard STRC method arising from second order STRC components. **(B)** Shows the difference between actual and estimated spike time differences in the 2-UCI network. A linear fit approximately describes the relationship between $\bar{\delta t}$ and δ*t*_*s*_ as: $\bar{\delta t}=-0.035064^{*}\delta t_{s}+2.2955$.

In order to characterize $\bar{\delta t}$ as a function of δ*t*_*s*_, we need to observe the 2-UCI network in 1:1 synchrony across a range for δt^*^ and δ*t*_*s*_. Since, δ*t*_*s*_ and δt^*^ are spike time differences of the theoretical map solution and simulations, respectively for a particular configuration of the 2-UCI network in 1:1 synchrony, they cannot be arbitrarily predetermined. Instead, by varying the temporal heterogeneity parameter *H* (25 ≤ *H* ≤ 45), we can cause the 2-UCI network to exhibit a range of 1:1 phase-locked synchrony states. Each of these 1:1 synchrony states allows us to measure the true spike time differences, δt^*^, from simulations and the theoretically estimated spike time difference, δ*t*_*s*_, between the two neurons. By noting δ*t*_*s*_, δt^*^, and the corresponding error $\bar{\delta t}$ for every value of *H* that produces 1:1 in-phase synchronization in the 2-UCI network, we were able to plot the error ($\bar{\delta t}$) as a function of δ*t*_*s*_ (see Figure 2A). We noticed that the relationship between $\bar{\delta t}$ vs. δ*t*_*s*_ is approximately linear and can be approximated via a linear fit as $\bar{\delta t}=-0.035064\times \delta t_{s}+2.2955$. This fitted line allowed us to calculate the approximate correction factor that has to be added to a given δ*t*_*s*_ in order for the STRC based solution to match simulation results (see Figure 2B). By generalizing δ*t*_*s*_ to any perturbation δ*t*, the correction factor can be expressed as $\bar{\delta t}=-0.035064\times \delta t+2.2955$. The correction factor ($\bar{\delta t}$) then lets us continue expressing the STRC as the linear sum of its first and second order components:

(9) $$\Phi_{i\infty }(g,\delta t^{*})=\Phi_{i1}(g,\delta t^{*})+\Phi_{i2}(g,\delta t^{*})$$

(10) $$\delta t^{*}=\delta t+\bar{\delta }t=0.964936*\delta t+2.2955$$

We will assume that all further references to STRC's in this paper incorporate the correction term δt^*^ that is calculated from the given δ*t*.

### 2.3. Spike timing dependent plasticity rule

**Figure 3:**
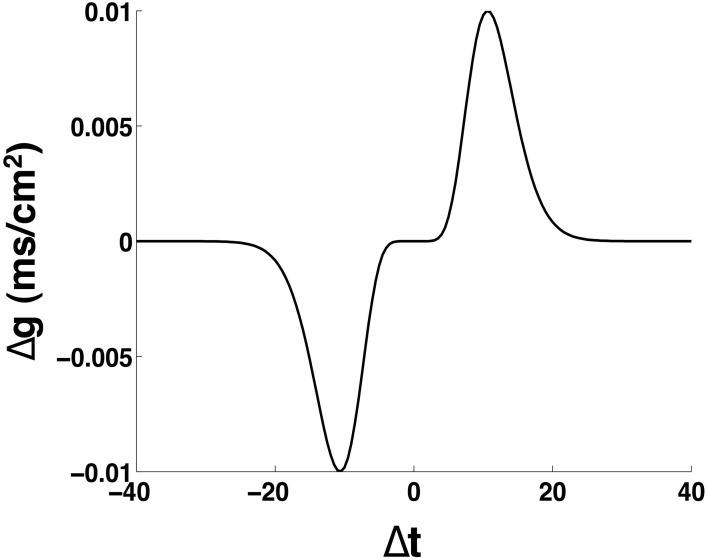
**Schematic of the spike timing dependent plasticity rule**. The parameters are: α = 0.94, β = 10, *g*_+_ = *g*_−_ = 0.01 mS/cm^2^.

We will consider the spike timing dependent plasticity (STDP) rule for GABAergic synapses, identified at GABAergic synapses in the entorhinal cortex (Haas et al., 2006), which is similar to the well-known STDP rule for excitatory synapses (Bi and Poo, 1998). The STDP rule is asymmetric with long-term potentiation for positive time intervals, Δ*t*, between the timing of post-synaptic neuron firing and the pre-synaptic neuron firing and long-term depression for Δ*t* < 0. We assume Δ*t* to be positive if the post-synaptic spike occurs after a pre-synaptic spike. The empirical function defining the STDP rule is given as:

(11) $$\begin{array}{l} \Delta g(\Delta t)=g\;+\;\tilde{g}(\Delta t)\text{ if}\Delta t>0\\ \;=g\;-\;\tilde{g}(\Delta t)\text{ if}\Delta t\langle 0 \end{array}$$

where $\tilde{g}\left(\Delta t\right)=\frac{1}{\beta^{\beta }e^{-\beta }}\alpha^{\beta }|\Delta t|\Delta t^{\beta -1}e^{-\alpha |\Delta t|}$ and *g*_+_ ≥ *g*_−_. The parameters are β = 10 and α = 0.94, which provide a temporal window of ±20 ms over which the efficacy of the synaptic plasticity is non-zero (Haas et al., 2006). Figure 3 presents the learning rule we use for STDP. In all the results presented here, unless otherwise mentioned, we set *g*_+_ = *g*_−_ = 0.01 and we assume an additive (linear) update rule for the modification of the synaptic strengths in the network (Caporale and Dan, 2008). Furthermore, we implement the STDP rule through all-to-all interactions, whereby after a spike occurs in a given post-synaptic neuron all the synapses from pre-synaptic neurons with immediately preceding spikes are potentiated whereas all the synapses from pre-synaptic neurons with spikes occurring after the post-synaptic spike are depressed (Bi and Wang, 2002; Froemke and Dan, 2002; Froemke et al., 2006).

### 2.4. Synchronization metric

We will measure the degree of synchronization *S* in the N-MCI networks by quantifying the amount of fluctuations in the network activity (Hansel and Sompolinsky, 1992; Kudela et al., 2003). Given the values for the membrane potentials *V*_*i*_(*t* − *kt*_*s*_) where *i* = (0, 1, ⋯*N* − 1), *k* = (0, 1, ⋯*M* − 1), *t*_*s*_ is the sampling time interval and *M* quantifies the time horizon *T* = *Mt*_*s*_, we define:

(12) $$S(N)=\frac{N\sigma_{V}}{\sum_{i=0}^{N-1}\sigma_{V_{i}}}$$

where $\sigma_{V}^{2}$ represents the variance in the mean field activity of the network and $\sigma_{V_{i}}^{2}$ represents the variance in the membrane potential of ith neuron in the network measured over a large time interval *T*. If we define $\langle X\rangle_{t}=\frac{1}{T}\int_{t-T}^{t}X\left(t\right)dt$, then σ_*V*_ and σ*_V_i__* are given as:

$$\begin{array}{l} \sigma_{V_{i}}^{2}={\langle V_{i}^{2}\rangle }_{t}-{\langle V_{i}\rangle }_{t}^{2}\\ \sigma_{V}^{2}={\langle {\left(\frac{1}{N}\sum_{i}V_{i}\right)}^{2}\rangle }_{t}-{\langle \frac{1}{N}\sum_{i}V_{i}\rangle }_{t}^{2} \end{array}$$

The properties of *S* are well-characterized in literature (Golomb and Rinzel, 1993). *S* essentially measures the average fluctuations of the mean membrane voltage of the network over a long time duration. It is normalized by the sum of individual neuron membrane fluctuations such that *S* scales between 0 and 1. In particular, for a fully synchronized network such that *V*_*i*_(*t*) = *V*(*t*) ∀*i* ∈ [0, *N* − 1], *S* = 1, whereas 0 ≤ *S* < 1 in all other situations.

## 3. Results

### 3.1. Synchronization manifold of the 2-MCI network

**Figure 4:**
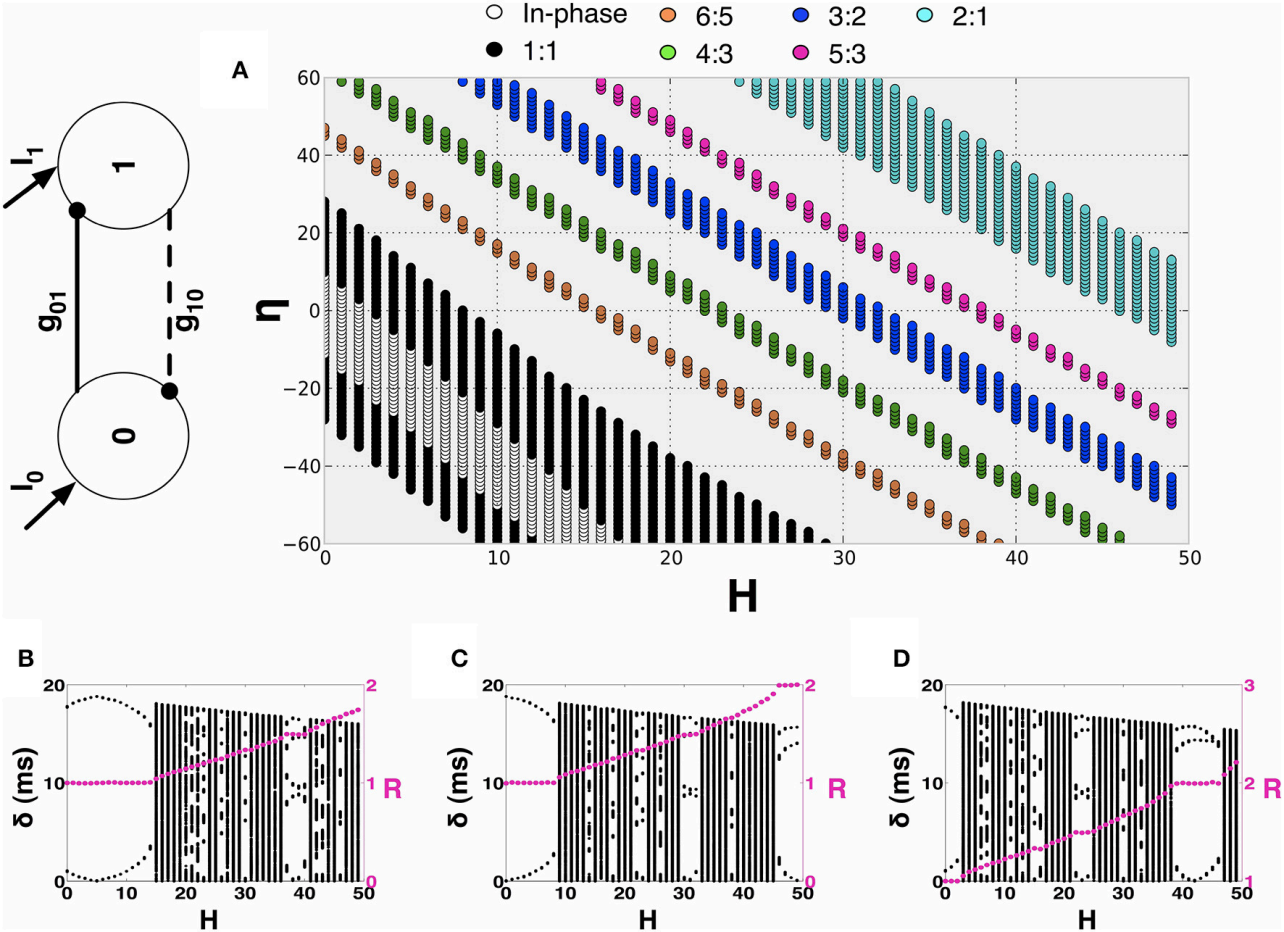
**Synchronization manifold of the 2-MCI network. (A)** Synchronization manifold of the 2-MCI network (shown in inset). The Figure shows the domain of m:n synchronization for the 2-MCI network in the two dimensional plane spanned by the network heterogeneity parameter H, and the network imbalance parameter η. In **(B–D)**, we plot the time lag δ between the firing times of the two neurons in the 2-MCI network as function of the network temporal heterogeneity parameter *H* for three different cases of the network temporal heterogeneity parameter η = {−20, 0, 20}, respectively. In each Figure, we also plot the ratio of mean firing periods of the two neurons *R* (magenta dots).

In this section, we investigate the structure of the synchronization manifold of the 2-MCI network as a function of the network heterogeneity parameters: {*H*, η}. We emphasize that all of our results critically depend on the structural heterogeneity parameter, η (Equation 4) and on the temporal heterogeneity parameter *H* (Equation 2). Now, let *t*_0, *i*_ (*i* = 1, 2, ⋯ ) and *t*_1, *j*_ (*j* = 1, 2, ⋯ ) represent the spike times for the two coupled neurons 0 and 1 in the 2-MCI network (Figure 4A). We define the mean period $\langle T_{X}\rangle =\underset{n\to \infty }{\lim}\frac{t_{X,n}}{n}$ (*X* = 0, 1) and the time lag δ_*n*_ = *t*_1,*n*_ − *t*_0,*m*_ between the nearest spike times of the two neurons. The index *m* corresponds to the mth spike from neuron 0 such that *t*_1,*n*−1_ ≤ *t*_0, *m*_ ≤ *t*_1,*n*_. For a given value of *H* and η, we numerically solve Equation (1) in order to estimate 〈*T*_0_〉 and 〈*T*_1_〉. If there exists *m, n* ∈ ℕ and *m* ≠ 0 and *n* ≠ 0 such that the ratio:

(13) $$R=\frac{\langle T_{0}\rangle }{\langle T_{1}\rangle }=\frac{m}{n}$$

we label the point {*H*, η} on the two dimensional hyper-plane spanned by *H* and η as the m:n synchronization point. Hence R describes the order of a synchronized pair of neurons as the ratio of their firing periods. The results of these calculations for 0 ≤ *H* < 50 and −50 ≤ η < 50 are summarized in Figure 4A. For example, the black dots in Figure 4A span the region of 1:1 synchronization. In this situation δ_*n*_ approached a fixed value δ_*s*_ ≤ 〈*T*_*X*_〉 representing a phase-locked state (see Figures 4B–D). The regions of higher order synchronization are depicted in various colors as follows: *R* = 6:5 (in orange); *R* = 4: 3 (in green); *R* = 3: 2 (in blue); *R* = 5: 3 (in magenta); and *R* = 2: 1 (in cyan). An important feature of the structure of the synchronization manifold is that the range of values of dynamical heterogeneity *H* over which the 2-MCI network can sustain 1:1 synchronization is significantly enhanced as the value of structural heterogeneity η decreases below zero. In other words, the effect of increasing *H*, resulting in an increase in the mismatch between the intrinsic firing rates of the two coupled neurons, can be compensated by increasing the strength (*S*) and simultaneously decreasing the sensitivity (*K*) of the neuron with low intrinsic firing rate. Yet another feature of the synchronization manifold is that the minimum absolute value of structural heterogeneity |η| required to sustain 1:1 synchronization in the 2-MCI network increases monotonically with increasing heterogeneity *H*. Furthermore, islands of higher order synchronization states emerge as *H* increases for a given fixed value of η. We will see in Section 3.2, this property of the synchronization manifold will significantly influence the evolution of the 2-MCI network under the influence of STDP.

In Figures 4B–D, we plot δ_*n*_ as function of *H* for the cases η = {−20, 0, 20}, respectively. We also plot *R* (magenta dots in Figures 4B–D, y-axis on the right) as a function of H. We see from Figure 4C that for a perfectly homogeneous 2-MCI network, i.e., *H* = η = 0, the two coupled neurons exhibit 1:1 in-phase synchronization. In the absence of structural heterogeneity (η = 0), the network can sustain 1:1 synchronization for 0 ≤ *H* < 8. However, as can be seen from Figure 4A, for η = −20, the range of values for *H* over which the network can sustain 1:1 synchronization is enhanced to 1 < *H* < 15. When η < −30, we note that the range of *H*-values for 1:1 synchronization does not include *H* = 0, which suggests that the structural heterogeneity is such that the network tends to exhibit master-slave dynamical structure wherein the influence of one neuron (neuron 0 in this case) on to the target neuron (neuron 1 in this case) is much stronger than in the vice versa case. In the case when η = 20, the bias toward master-slave dynamics is significantly stronger with neuron 1 being the driver neuron and neuron 0 being the slave neuron. This is because, not only is neuron 1 firing at an intrinsically greater rate than neuron 0, but also the synaptic drive onto neuron 0 is stronger, which further slows the firing rate of neuron 0. The network is therefore unable to exhibit 1:1 synchronization. At the same time, the domain of higher order synchronization such as 2:1 synchronization is significantly enhanced.

In summary, the key conclusion from this analysis is that the 2-MCI network can sustain 1:1 synchronization for a wide range of heterogeneity in the intrinsic firing rates of the coupled neurons for an appropriate choice of structural heterogeneity in the network, i.e., by increasing strength of neuron 0 relative to the strength of neuron 1 and decreasing the sensitivity of neuron 0 relative to that of neuron 1, to trigger an effective structural imbalance η < 0.

### 3.2. STDP induced emergent synchronization in 2-MCI network

**Figure 5:**
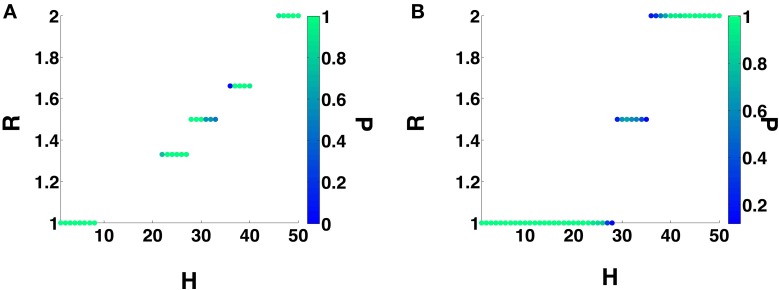
**2-MCI Synchronization Ratio probability for η_0_ = 0. (A)** Shows the probability of the 2-MCI network to reach different orders of synchronization without STDP. **(B)** Shows the probability for different orders of synchronization with STDP. The range of *H* for which the network can synchronize is significantly enhanced for the 1:1 domain and higher orders.

We will now investigate the effect of STDP on the dynamics of a heterogeneous 2-MCI network. For all the results presented in this section, we will begin with a structurally homogeneous network i.e., initial η = η_0_ = 0 and investigate the influence of STDP on the network dynamics for various values for *H*. For a given value of 0 ≤ *H* < 50, we numerically solve Equation (1) beginning with a random set of initial conditions *V*_*i*_ (*i*= 0, 1) in the range [−70, −50] mV. We perform simulations (each simulation run is for 5 s) for each of the two cases: (1) no STDP learning, with static synaptic conductance values *g*_01_ = *g*_10_ = *g*_0_∕2 and (2) with STDP learning, following the additive rule given in Equation (11). The initial synaptic conductance values are set to be the same as for the static case. For each simulation run, after the network has evolved into an asymptotic state (asynchronous firing or the state of m:n synchronization) we estimate the ratio *R* (see Equation 13). The results of these calculations are summarized in Figure 5, where we plot *R* as function of *H* and the color code represents the fraction *P* of the simulations generating a given value of R. For example, from Figure 5A, we see that the 2-MCI network exhibits a stable state of 1:1 synchronization for 0 ≤ *H* < 9. The network also exhibits narrow bands of stable higher order synchronization for larger heterogeneity values (as seen in Figure 4A). It is clear from Figure 5A that many of these states of stable synchrony are global attractors for the network, i.e., the state of stable synchronization is achieved independent of the initial conditions (*P* = 1) on the membrane potentials of the neurons in the network.

#### 3.2.1. STDP induced 1:1 synchronization

**Figure 6:**
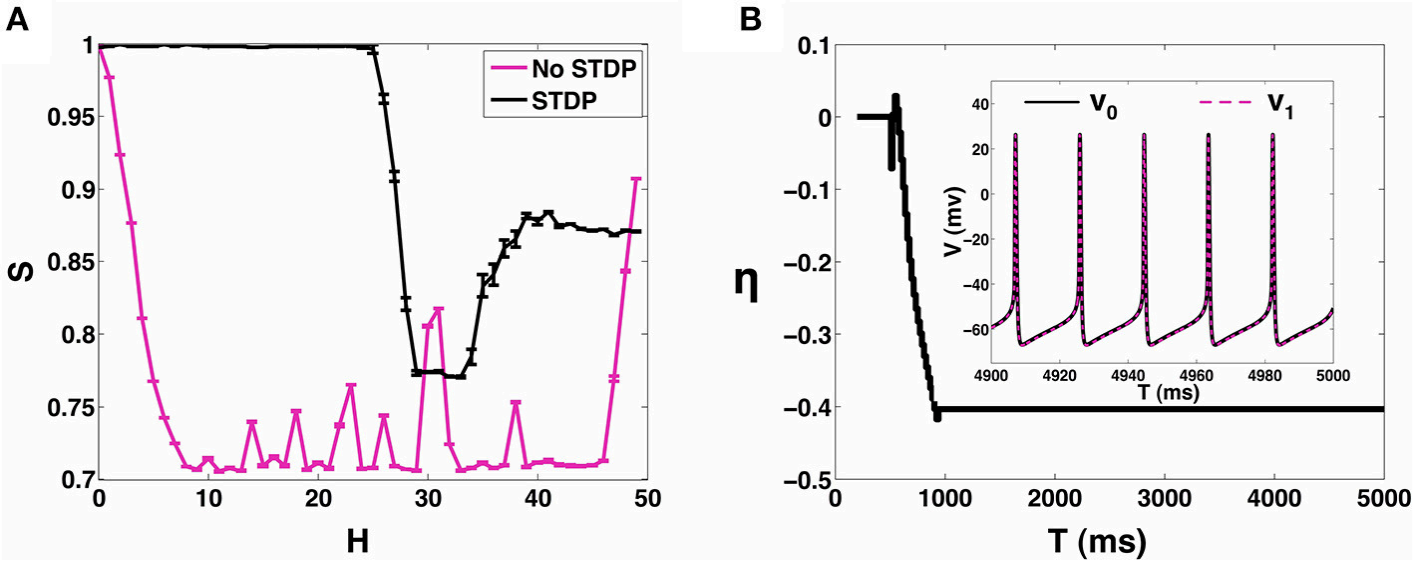
**2-MCI Synchrony metric. (A)** A measure of the synchrony metric when STDP is enabled (black) and without STDP (magenta) for the range 0 ≤ *H* ≤ 50. **(B)** Shows the evolution of η for an example initial case of {η_0_ = 0, *H* = 10}. In the inset we show the corresponding voltage traces of the neurons after reaching in-phase synchronization.

In the presence of STDP, the synaptic coupling strengths evolve such that, the range of heterogeneity values over which the network can sustain stable states of 1:1 synchronization (*P* = 1) is enhanced to include the range 0 ≤ *H* < 24 (see Figure 5B). In Figure 6A, we plot the mean value (and the standard error) of synchronization metric *S* as function of *H*. We note that *S* = 1 for the range of *H*-values over which the network can sustain a globally stable attractor state of 1:1 synchronization, representative of the case when the network is fully synchronized (i.e., 1:1 in-phase synchronization). As noted in Section 3.1, in absence of STDP, only the homogeneous 2-MCI network can exhibit fully synchronized state (also see the black curve in Figure 6A). Finally, as can be seen from Figure 5A, while the likelihood for the 2-MCI network to exhibit 1:1 synchronization is zero (*P* = 0) for *H* > 8, with STDP learning (Figure 5B) the likelihood for the 2-MCI network to sustain 1:1 synchronization, *P* ≠ 0, for the range of 0 ≤ *H* < 27, albeit the likelihood for the network to sustain 1:1 synchronization decreases with increasing *H*.

In order to understand how the structural properties of the 2-MCI network change as the network evolves to a state of 1:1 in-phase synchronization under the influence of STDP, in Figure 6B, we plot the time evolution of η for the case *H* = 10. The inset of the Figure shows a sample time-trace of the membrane potential of the two neurons after the network has evolved into the asymptotically stable state of 1:1 in-phase synchronization. The network is initially structurally symmetric, i.e., η_0_ = 0. The STDP learning begins at time *t* = 200 ms, immediately after which, the network imbalance begins to evolve ultimately reaching a steady state value η ≈ −40.

Starting from η_0_ = 0, the network evolves vertically downwards in terms of the two-dimensional plot of the synchronization manifold in Figure 4A, such that the synaptic strength of the slower firing neuron is enhanced while at the same time its sensitivity is decreased. As noted earlier (Section 3.1), this is the type of structural heterogeneity required for the 2-MCI network to sustain 1:1 synchronization for *H* > 8. This evolution of the 2-MCI network to a preferred state of 1:1 in-phase synchronization is critically dependent on the form of the learning rule that corresponds to Δ*g* > 0(< 0) for Δ*t* > 0(< 0), respectively. This form of the learning rule implies that for a given synapse, every time the post-synaptic neuron fires, the strength of synapse will increase where as every time the pre-synaptic neuron fires, the synaptic strength will decrease. For the configuration of the 2-MCI network with *H* = 20, neuron 1 has a greater intrinsic firing rate than neuron 0 such that in absence of STDP, on average 〈*T*_1_〉 < 〈*T*_0_〉. Thus, after STDP is active, the synapse *g*_01_ (with neuron 1 being the post-synaptic neuron for the synapse) increases more often than it decreases and consequently η fluctuates with an increased bias toward η < 0 until the network evolves into the domain of 1:1 synchronization (see Figure 4A) at which point 〈*T*_1_〉 = 〈*T*_0_〉. Since Δ*g* ≠ 0 for Δ*t* ≠ 0, the network continues to evolve toward the fixed point δ = 0 corresponding to the stage where η monotonically decreases (Figure 6B) until the network reaches a stable state of 1:1 in-phase synchronization. Hence in the case of 1:1 synchrony, STDP evolves the structural heterogeneity (η) such that it compensates for any temporal heterogeneity (*H* > 0).

#### 3.2.2. STDP induced m:n synchronization

We can see from Figure 5B that for *H* > 24, under the influence of STDP, the state of 1:1 synchronization is not a global attractor of the 2-MCI network, i.e., *P* < 1. The network can evolve to states of higher order synchronization depending on the initial conditions on the membrane potentials of the neurons. It is also clear from Figure 5B that the likelihood (i.e., the fraction of simulation runs) for the network to evolve to a state of higher order synchronization increases with increasing *H*. For example, for *H* > 45, the probability for the network to evolve to a state of stable 2:1 is 1.

The synchronization manifold of the static 2-MCI network (Figure 4A) provides an insight into the reason for 1:1 synchronization not being the global attractor of the 2-MCI network under the influence of STDP learning. As discussed in Section 3.2.1, the STDP rule modulates the synaptic strengths such that the 2-MCI network evolves from a structurally balanced state of η_0_ = 0 toward a structurally imbalanced state with η < 0 in order to compensate for *H* ≠ 0. However, for *H* ≥ 24, the synchronization manifold of the 2-MCI network exhibits multiple domains of higher order synchronization states for η < 0 and there is a likelihood for the network to enter these domains of higher order synchronization before the network can reach the state of 1:1 synchronization. As *H* increases beyond *H* = 18, the number of higher order synchronization states increase and at the same time the domain of 1:1 synchronization moves further away from the balanced state of η_0_ = 0 and correspondingly the likelihood of the network to evolve to 1:1 synchronization decreases, as is evidenced from Figure 5B.

#### 3.2.3. Analysis of emergent 1:1 in-phase synchrony in the 2-MCI network

In this section, we analyze the stability of the emergent 1:1 in-phase synchronization in the 2-MCI network under the influence of STDP by deriving a nonlinear map for the evolution of the time lag δ between successive spike times of the two neurons in the 2-MCI network using the framework of STRC's. We consider the specific case of the network configuration with parameters: *H* = 10 and η_0_ = 0. Following from the results presented in Section 3.1, for the choice of network heterogeneity and imbalance parameters, in the absence of STDP, the two neurons in the 2-MCI network will fire asynchronously, with mean firing rate of neuron 1 greater than that of neuron 0. Invoking STDP in this situation will, on average, cause the strength *S*_0_ of neuron 0 to increase, while at the same time it will cause the sensitivity *K*_0_ of neuron 0 to decrease. This is because, more often than not, the fast firing neuron 1 will emit more than one spike during each period of spiking from neuron 0. Each firing of neuron 1 (the post-synaptic neuron to synapse from neuron 0 to neuron 1), in turn, increases the strength of synapse *g*_01_ through the STDP rule. By the same token, the strength of synapse *g*_10_ will decrease creating an effective network imbalance η < 0. Thus, starting from a structurally balanced network *I*_net_ = 0, the network will evolve vertically downwards in the two dimensional η − *H* plane. As seen from Figure 6, for the specific case of *H* = 10 beginning with η_0_ = 0, the network will eventually evolve into the domain of 1:1 synchronization.

**Figure 7:**
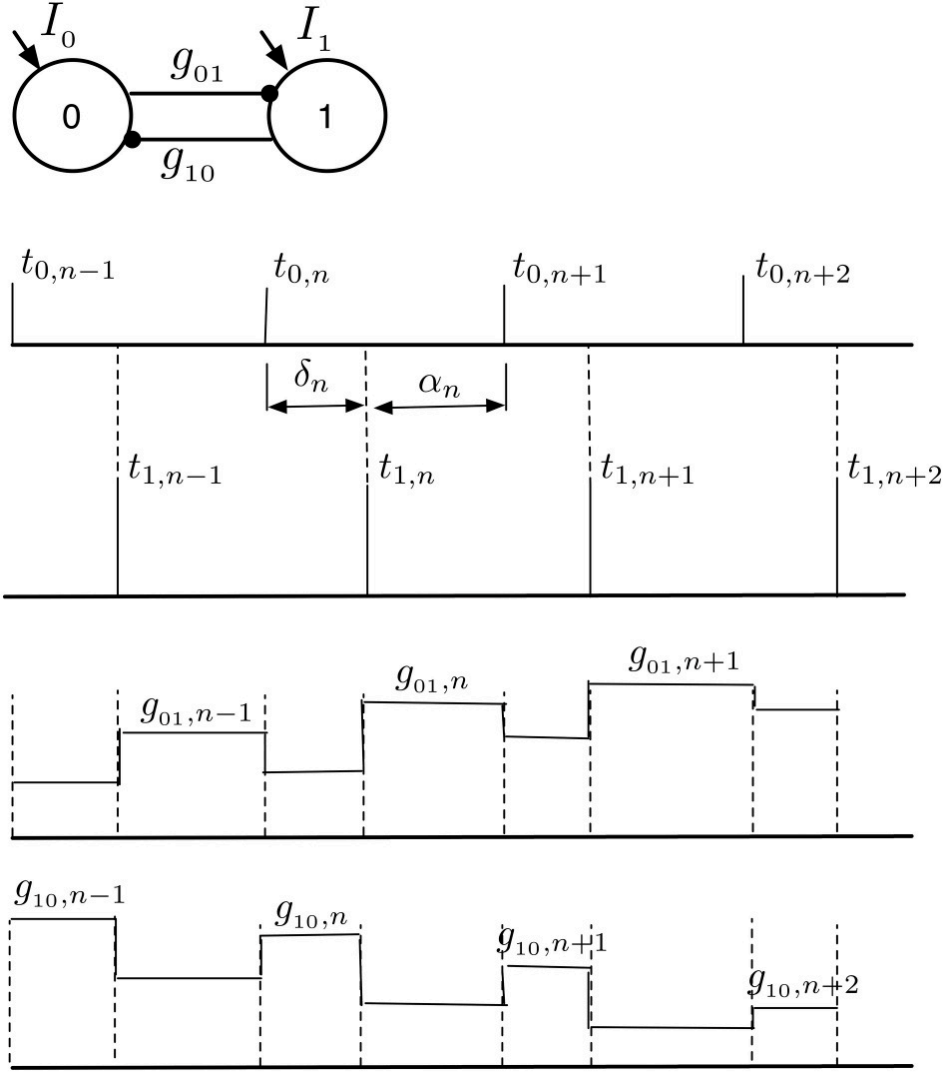
**1:1 Phase locked synchrony in a 2-MCI network**. This figure illustrates the evolution of consecutive spike times {δ, α} during phase locked 1:1 synchronization. The corresponding changes in {*g*_01_, *g*_10_} due to the STDP rule are also shown. We use this concept of 1:1 phase locked spike-time and STDP evolution to derive the map defined in Equation (15).

In order to understand how the synaptic strengths evolve within the domain of 1:1 synchronization to the final stable state of 1:1 in-phase synchronization, we use the mathematical framework of STRC's to derive a nonlinear map for the evolution of the time-lag δ and the synaptic strengths *g*_*ij*_ (*i, j* = {0, 1}). We assume that once the network enters the phase locked state of 1:1 synchronization with 〈*T*_0_〉 = 〈*T*_1_〉, the phase locked state remains quasi-static as the synaptic strength continues to evolve toward the asymptotic state of 1:1 in-phase synchronization. In Figure 7, we present a snapshot of sequence of spikes from the two neurons after the neurons have entered the domain of 1:1 synchronization. Following from Figure 7, if *t*_0,*n*_ and *t*_1,*n*_ represents the timing of nth spike originating from neuron 0 and neuron 1 in the 2-MCI network, respectively, then the subsequent spike from the two neurons will emerge at times: *t*_0,*n*+1_ and *t*_1,*n*+1_ such that:

(14) $$\begin{array}{l} \;t_{0,n+1}=t_{0,n}+T_{00}\left[1+\Phi_{0\infty }\left(g_{10,n},(t_{1,n}-t_{0,n})\right)\right]\\ \;t_{1,n+1}=t_{1,n}+T_{10}\left[1+\Phi_{1\infty }\left(g_{01,n},(t_{0,n+1}-t_{1,n})\right)\right]\\ g_{01,n+1}={\tilde{g}}_{01,n}+\Delta g(t_{1,n+1}-t_{0,n+1})\\ g_{10,n+1}={\tilde{g}}_{10,n}+\Delta g(t_{0,n+1}-t_{1,n}) \end{array}$$

where ${\tilde{g}}_{01,n}=g_{01,n}+\Delta g\left(t_{1,n}-t_{0,n+1}\right)$ and ${\tilde{g}}_{10,n}=g_{10,n}+\Delta g\left(t_{0,n}-t_{1,n}\right)$, respectively. In writing Equation (14), we assume that *g*_*ij, n*_ is the conductance of synapse from the pre-synaptic neuron i to the post-synaptic neuron j, immediately after nth spike is emitted from the post-synaptic neuron. We further assume that STDP induced change in synaptic conductance is instantaneous. The nonlinear map for the evolution of the time-lag δ_*n*_ = *t*_1,*n*_ − *t*_0,*m*_ (index m corresponds to the mth spike from neuron 0 such that *t*_1, *n*−1_ ≤ *t*_0, *m*_ ≤ *t*_1, *n*_) and the synaptic conductances *g*_*ij, n*_ can be obtained from Equation (14) as:

(15) $$\begin{array}{l} \;\alpha_{n}=T_{00}\left[1+\Phi_{0\infty }(g_{10,n},{\tilde{\delta }}_{n})\right]-{\tilde{\delta }}_{n}\\ \;\delta_{n+1}=T_{10}\left[1+\Phi_{1\infty }(g_{01,n},{\tilde{\alpha }}_{n})\right]-{\tilde{\alpha }}_{n}\\ g_{01,n+1}=g_{01,n}+\Delta g(-\alpha_{n})+\Delta g(\delta_{n+1})\\ g_{10,n+1}=g_{10,n}+\Delta g(-\delta_{n})+\Delta g(\alpha_{n}) \end{array}$$

where $\tilde{\delta }=\delta \;\mathrm{mod}\;T_{00}$ and $\tilde{\alpha }=\alpha \;\mathrm{mod}\;T_{10}$.

To determine whether Equation (15) can predict 1:1 in phase synchronization for the 2-MCI network under the influence of STDP learning, we apply the discrete map for the specific cases of the 2-MCI network with heterogeneity *H* = 10 and *H* = 20 with initial conditions *g*_01, 0_ = *g*_10, 0_ = *g*_0_∕2 corresponding to the case η_0_ = 0 and δ_0_ = 0.

**Figure 8:**
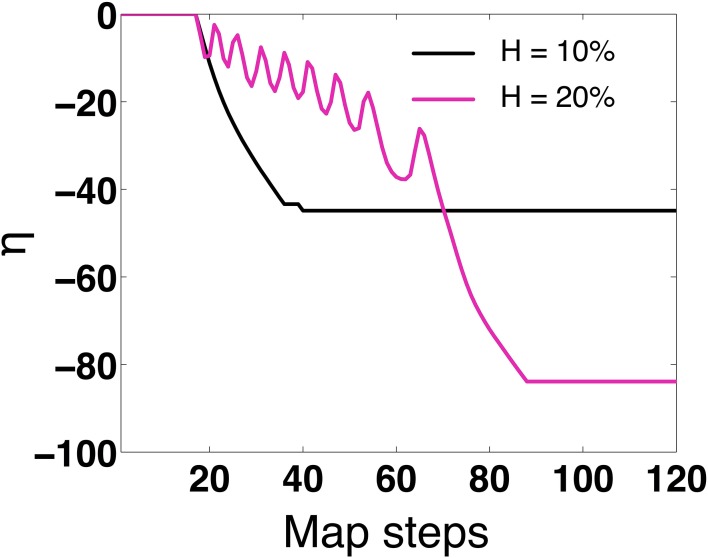
**Map evolution of η**. We validate that under STDP, the map evolves to stationary values of η that allow in-phase synchronization. For *H* = 10 the map predicts η = −44 (as compared to η = −40 for 2-MCI simulations). For *H* = 20 the map predicts η = −82 (as compared to η = −80 for 2-MCI simulations).

The results are presented in Figure 8. For the case *H* = 10, we see from Figure 8 that η evolves to a steady state value of η = −44, with δ_*s*_ = 18.8, α_*s*_ = 0.1 ms, *g*_01_ = 0.073 mS/cm^2^ and *g*_10_ = 0.027 mS/cm^2^. The mean period of the synchronized network (described in Section 3.1) is 〈*T*_0_〉 = 〈*T*_1_〉 = 18.9 ms. For the special case of 1:1 synchrony, we will refer to the network period as:

(16) $$\langle T_{0/1}\rangle =\langle T_{0}\rangle =\langle T_{1}\rangle$$

Upon simplifying (Equation 15) for the 1:1 steady-state, one can derive a straightforward relationship between δ_*s*_ and 〈*T*_0∕1_〉 as: 〈*T*_0∕1_〉 = δ_*s*_ + α_*s*_. We see that when α_*s*_ ≈ 0 then 〈*T*_0∕1_〉 = δ_*s*_ which is the in-phase 1:1 solution. Though the discrete map correctly predicts the existence of a steady state fixed point solution, a minor discrepancy in the discrete map solution for steady state value of η exists. There is a difference in η of ≈4 between the map's solution and to the solution obtained by numerically solving Equation (1) under the influence of STDP. This discrepancy stems from the linear approximation of the correction factor $\bar{\delta t}$ as a function of δ*t*, as described in Section 2.2 regarding STRC's.

For the case *H* = 20, the steady state solution for the discrete map evolves to an in-phase solution at η = −82. As *H* approaches 27 and η = 0, the map fails to evolve to a steady state solution as expected. The map cannot accurately predict higher order synchronization, as Equation (15) is specifically derived for the 1:1 phase locked synchronization domain. From these findings we conclude that the discrete map for the temporal evolution of the spike time lag δ and the structural heterogeneity η is able to correctly predict the existence of a stable fixed point solution for 1:1 synchronization of the 2-MCI network under the influence of STDP learning.

We next determine if the 1:1 in phase is stable. First, we performed linear stability analysis of the discrete map in Equation (15) for the fixed point in-phase solutions {δ_*s*_, α_*s*_, *g*_01*s*_, *g*_10*s*_} = {18.8, 0.1, 0.073, 0.027}. We find that all the eigenvalues are real and {λ_δ_, λ_α_} < 1, indicating stability. However, {λ_*g*_01__, λ_*g*_10__} = 1. These eigenvalues suggest that while the in-phase solution is stable for static synapses, with STDP the system may be marginally stable or unstable. In order to determine if the system was marginally stable at the in-phase solutions, we examined the Jordan form of the linearized state matrix and observed that the Jordan blocks corresponding to unit eigenvalues were scalar. This served as theoretical confirmation for local stability of the system with STDP present.

**Figure 9:**
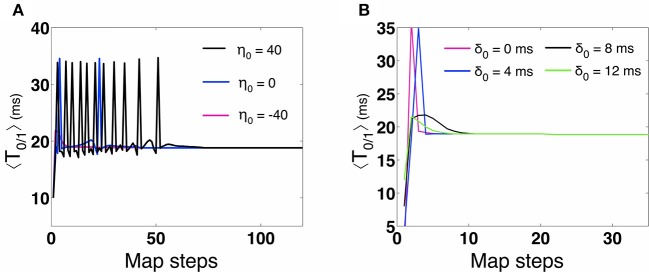
**Numerical stability analysis of the 2-MCI map. (A)** The evolution of the map's predicted synchrony period 〈*T*_0∕1_〉 for different initial conditions of η_0_. **(B)** The evolution of the map's synchrony period 〈*T*_0∕1_〉 for different initial conditions of δ.

Additionally, we numerically analyzed the sensitivity of the in-phase solutions reached through STDP. We tested the sensitivity of the solutions by varying the initial conditions of the state variables in Equation (15). In Figure 9A we show the evolution of the 1:1 in-phase map period of the 2-MCI network: 〈*T*_0∕1_〉 = δ_*S*_ for different initial values of η_0_ and *H* = 0. We see that for η_0_ = 0 and even η_0_ = 40, 〈*T*_0∕1_〉 evolves to the stable in-phase period of 18.9 ms, which is the exact in-phase period predicted by the 2-MCI simulations. The map stably evolves to the in-phase solution even for an initial value that is 200% greater than the solution point. We also confirm that 〈*T*_0∕1_〉 evolves to the in-phase period for different initial spike time lag (δ_0_) values of {0, 4, 8, 12} ms. This is illustrated in Figure 9B. These numerical analyses confirm that the in-phase state of the 2-MCI system is indeed locally stable.

### 3.3. STDP induced emergent synchronization in 100-MCI network

**Figure 10:**
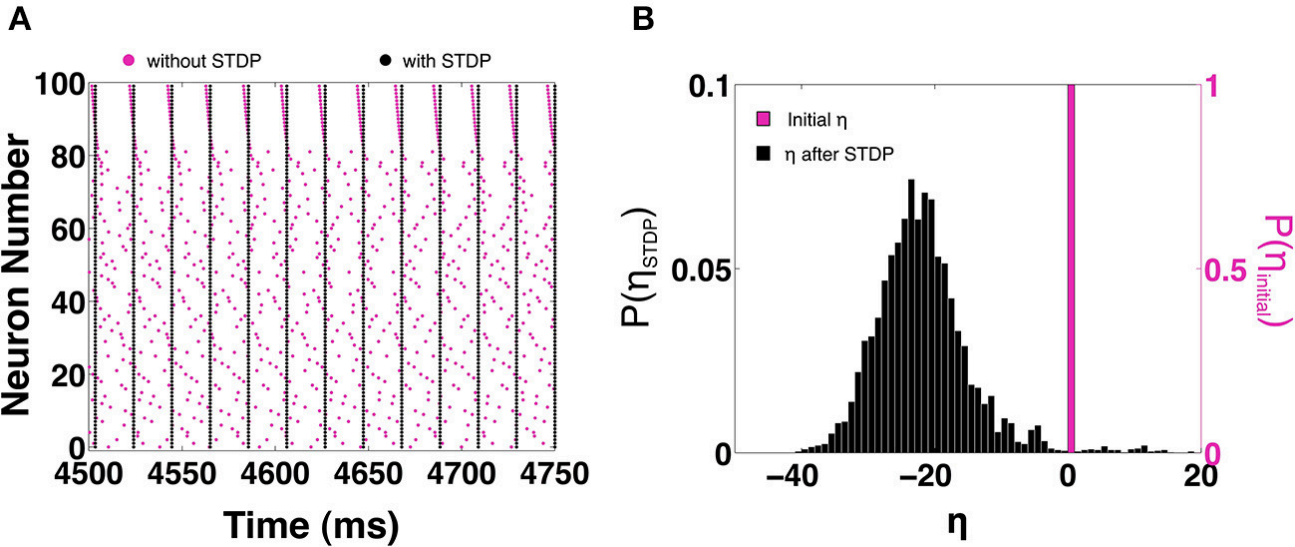
**STDP based synchronization in a 100-MCI network. (A)** Shows a raster of the spike times with STDP (black) and without STDP (magenta). **(B)** The corresponding distribution of η-values with STDP (black) and without STDP (magenta).

In this section we examine how STDP allows larger MCI networks to synchronize. We consider a homogeneous all-to-all coupled network of 100 neurons with temporal heterogeneity *H* = 10. In the absence of STDP, Figure 10A shows a raster of the spike times (magenta) when the network fails to synchronize. On enabling STDP, the network is able to reach perfect in-phase synchronization as shown by the black rasters in Figure 10A. In Figure 10B we show the fraction of η values before (magenta) and after STDP (black). We can see that STDP evolves the η-values toward a slightly positive-skewed Gaussian distribution with a mean of -20. As we have seen in the previous sections, STDP tends to evolve the synapses such that η decreases. As η decreases, the spike times become closer to each other (within the 1:1 synchronization domain). When η is sufficiently negative, the spike time differences are almost zero. This causes the STDP to suddenly stop evolving, causing the distribution of η to appear positively skewed.

**Figure 11:**
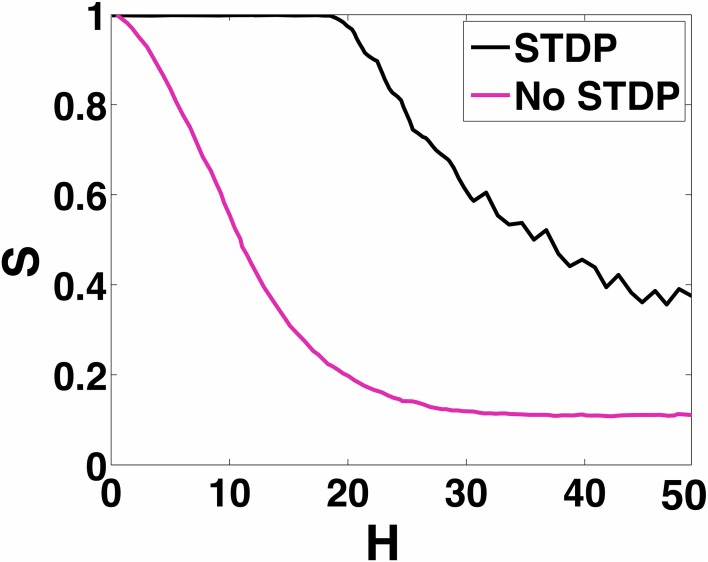
**100-MCI Synchrony metric**. Here we measure the measured synchrony of the 100-MCI network for a range of 0 ≤ *H* ≤ 50. In-phase synchronization is significantly enhanced for a wide range of 0 ≤ *H* ≤ 18 when STDP is enabled (magenta).

In Figure 11, we show how increasing *H* affects the ability of the 100 neuron MCI network to exhibit in-phase synchronization. In the absence of STDP, the synchronization measure *S* very quickly drops with increased *H*. However, when STDP is enabled, the network is able to sustain in-phase synchronization for *H* < 20. For *H* > 20, the network's synchronization measure gradually decreases.

**Figure 12:**
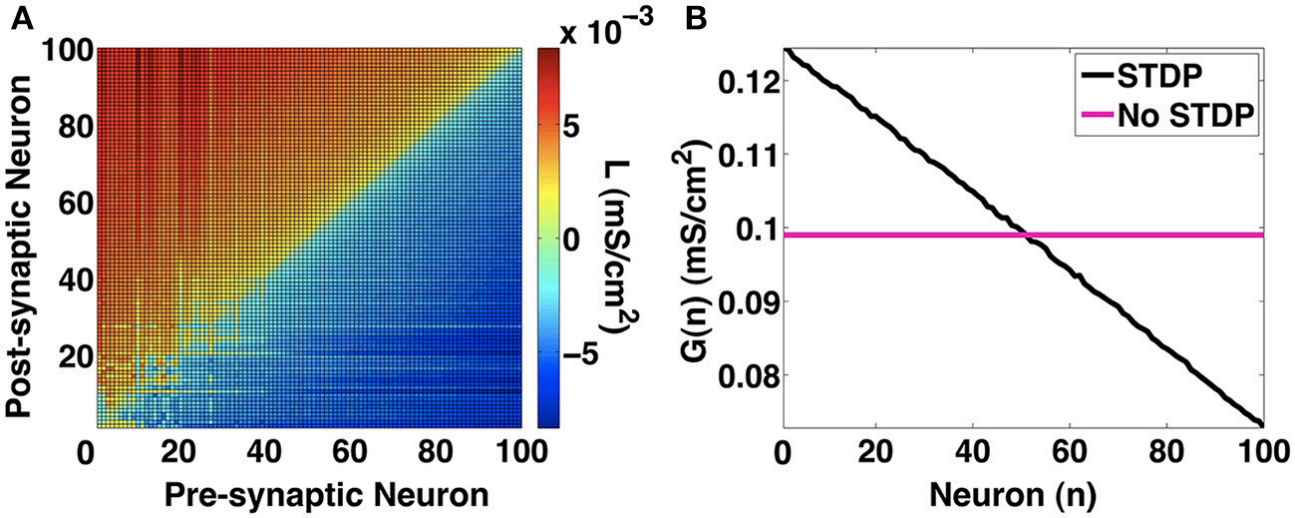
**Network metrics. (A)** Link imbalance of the 100-MCI network when *H* = 10. The synaptic strength differences between pairs of neurons are color coded. In general synapses originating from slower neurons (lower numbers on x-axis) have a positive network imbalance value. **(B)** The neuron strength metric measures the total outgoing synaptic strength for each neuron. With STDP, neuron strength linearly decreases in the direction of slowest firing neuron to the fastest neuron, indicating greater synaptic influences of slower neurons.

We also look at the other network metrics that we defined in the methods section. In Figure 12, we show the network link imbalance and neuronal strengths. The link imbalance measures the difference in synaptic strengths of coupled neurons in the network. It is expressed as a matrix *L*_*ij*_ = *a*_*ij*_*g*_*ij*_ − *a*_*ji*_*g*_*ji*_, where *i* and *j* refer to the pre-synaptic and post-synaptic neurons, respectively, and [*a*_*ij*_] ∈ [0, 1] refers to the connectivity between neuron *i* and *j*. For a network with homogenous synaptic strengths, *L* is a zero matrix. When the network evolves under STDP to in-phase synchronization, we notice in Figure 12A that the network imbalance shifts from a zero matrix to a skew symmetric matrix whose main diagonal is zero (indicating no self-synapses). For *i* < *j*, *L*_*ij*_ > 0 and for *i* > *j*, *L*_*ij*_ < 0. This indicates that STDP evolves such that the strength of synapses from slower neurons onto faster neurons are larger in value. This observation is further reinforced by the neuronal strength metric ($G_{i}=\underset{j}{\sum }a_{ij}g_{ij}$) shown in Figure 12B. Here, the sum of the strengths of outgoing synapses for every neuron is presented. Without STDP, all neurons project equal synaptic influences on each other. With STDP, as consistent with our earlier observations, the slowest neuron number 0 has the largest synaptic influence on the rest of the network while the fastest firing neuron number 100 has the weakest synaptic influence on other neurons. This shows that with STDP, the slower neurons can control the firing rate of the faster neurons thus increasing the chances of attaining synchronization. Our 100-MCI network investigations suggest that STDP is an effective mechanism that allows in-phase synchronization for a wide range of neuron firing rate heterogeneity. It also suggests that STDP induced synchronization may not be significantly affected by the scale of the MCI network.

## 4. Discussion

In this study we investigated if STDP in mutually coupled interneuronal networks can induce stable in-phase synchronization at gamma frequencies. We first investigated the domains of synchronization for a 2-MCI network in terms of structural and temporal heterogeneity, given by {η, *H*}. We identified the 1:1, in-phase and higher order synchronization domains for a static 2-MCI network. We noted that in general, as *H* increased, η had to decrease in order to maintain 1:1 and in-phase synchronization. The decrease in η corresponds to the case of *g*_01_ increasing while *g*_10_ decreases. This in turn, indicated that, for 1:1 and in-phase synchrony, the slower neuron 0, suppresses the faster neuron 1, such that their periods match. In the case of higher order synchronization such as 2:1 synchronization, the faster neuron fires more than once for every single time the slower neuron fires. Here, the period of the slower neuron is significantly increased due to suppression by the faster neuron.

Previous work by White et al. (1998) has shown that synchronization in MCI networks are susceptible to even slight increase in firing rate heterogeneity. Our group had shown that STDP was one way by which stable synchronous oscillations could be achieved in UCI and 2-MCI networks (Talathi et al., 2008). This was based on the findings of Haas et al. (2006), that reported STDP was present in inhibitory synapses in the second layer of the entorhinal cortex. Experimental measurements provided the STDP learning rule (Haas et al., 2006) that was used in computational experiments by Talathi et al. (2008). We demonstrated that in the presence of firing rate heterogeneity, STDP resulted in significant enhancement of network synchronization. A STRC based theoretical map was derived to verify the stability of UCI in-phase synchronization.

In other works, STRC's have been used to calculate future spike times based on periodic synaptic perturbations from other neurons. Most of these studies assumed that the STRC's displayed weak second order components. For example in the work done by Talathi et al. (2008), due to weak second and higher order STRC components, the total spike time change for periodic synaptic perturbations was represented as Φ_*i*∞_(δ*t, g*) = Φ_*i*1_(δ*t, g*) + Φ_*i*2_(δ*t, g*). Where, δ*t* was the time of perturbation after every spike in each firing cycle. In the course of our investigations we observed that a fast firing Wang-Buzsaki neuron (Wang and Buzsáki, 1996) with inhibitory synaptic input, produced STRC's with a significant second order component. This in turn indicated that calculating spike time shifts using the Φ_*i*∞_ term would lead to incorrect predictions.

Recently, Talathi et al. (2009) demonstrated an empirical approach for predicting spike times when higher order STRC's were present. However, this method was specific for shunting synapses. The approach used in this paper is more generic. We considered cases where a 2-UCI network exhibited synchrony for a certain set of parameters. Then the error between predicted spike times and actual spike times were noted, and an approximately linear relationship between perturbation time δ_*t*_ and error in spike-times $\bar{\delta_{t}}$ was observed. Using this relationship we derived a correction factor for the perturbation time, which when incorporated into STRC calculation, allowed the term Φ_*i*∞_ to more precisely describe spike time shifts.

In this work, we performed an in-depth analysis on the synchronization of 2-MCI network and eventually, a 100-MCI network. We utilized the same STDP learning rule form as used by Talathi et al. (2008). We also use a more biophysically relevant fast spiking interneuron model of Parvalbumin-expressing basket cells (Wang and Buzsáki, 1996). As in our previous work, the STDP mechanism implemented makes two assumptions: (1) Only the spike time difference between neighboring pairs of spikes was considered. (2) The effects of STDP modulation were linearly summed. These assumptions allowed us to obtain an analytical form of the STDP rule.

We began our study on the 2-MCI network by identifying the synchronization domain in terms of the structural and firing rate heterogeneity parameters {η, *H*}, respectively. We showed that decreasing η could compensate for increasing *H* (up to an extent), in terms of maintaining 1:1 and in-phase synchronization. We also identified higher order synchronization domains for the 2-MCI network. The synchronization manifold of the 2-MCI network provided us with an understanding of the direction in which η would have to evolve in a 2-MCI network, in order to attain 1:1 or in-phase synchronization, as *H* increased.

In the presence of STDP, for η_0_ = 0, the 2-MCI network was found to evolve to in-phase synchronization for a significant range of *H* < 25. We determined that for *H* > 25, STDP had a higher probability to evolve the network toward higher order synchronization. Regardless, STDP enhanced the domain of synchronization not only for 1:1 synchronization, but also for higher order synchronization. This also indicated that depending on the initial conditions of η_0_, STDP could variably evolve the network to different orders of synchrony.

In order to determine if the 1:1 and in-phase synchronization states of the network were stable, we mathematically analyzed the 2-MCI network and derived a STRC based map for predicting 1:1 and in-phase synchronization. We first validated the map by ensuring that the predicted spike time differences ({δ, α}) were the same as that of the static 2-MCI simulations, within the 1:1 synchronization domain. We then observed the map's evolution of η with STDP for an initial η_0_ = 0 and *H* = 10. We found that the predicted value of η for in-phase synchronization, was around 44 which was close to the 2-MCI STDP evolution of η = 40. The slight discrepancy in the values of η most likely arose from the linear approximation of the STRC correction term $\bar{\delta t}$ as a function of δ*t*_*s*_. Having validated that the map behaves closely to the 2-MCI simulations, we performed linear stability analysis on the discrete mathematical map expressed in Equation (15). Our findings indicated that the eigenvalues for the spike times {δ, α} were stable (< 1). However, the eigenvalues corresponding to the STDP evolution of {*g*_01_, *g*_10_} were marginally stable. To prove that system was at least locally stable, we performed further numerical analyses. Specifically, we examined the evolution of the map with STDP for different initial conditions of δ and η. We observed that the map's predicted 1:1 synchronization period (〈*T*_0∕1_〉 ≈ δ) and confirmed that it evolved to the period of the 2-MCI simulation in-phase synchronization, for a range of initial conditions. In fact η had to be increased significantly (η ≥ 20) beyond the stable value η = −40 in order for the map to fail to evolve to the in-phase synchronization regime.

We next examined how the scale of MCI networks affects STDP induced synchronization. We constructed and simulated a 100-MCI structurally homogeneous network (η_0_ = 0) with and without STDP for *H* = 10. In accordance with our previous results, we found that without STDP, the structurally homogeneous network was completely desynchronized. On enabling STDP, the 100-MCI network was able to exhibit in-phase synchronization for a significantly increased range of temporal heterogeneity *H* < 18. It must be noted that as η is only defined for any given pair of mutually coupled interneurons, in a larger 100-MCI network (without self synapses) the total number of synaptic pairs are 4950, and each pair has an associated η that describes the structural heterogeneity. We observed that with STDP, the distribution of η evolved toward a normal distribution with a slight positive skew. This small positive tail of η-values indicates that the distribution of η evolved toward the negative direction and ceased evolving when in-phase synchronization was reached.

All of our results suggest that STDP is a very viable mechanism for the formation of interneuronal gamma (ING) oscillations. The original firing rates of the free running interneurons (*H* = 0) are approximately 60 Hz. In the 2-MCI network, we observed that the firing rates were in the ranges of 52 and 53 Hz, for 1:1 and in-phase synchronization, respectively. The 100-MCI network was also able to sustain 50 Hz in-phase synchronization. These frequencies are well within the gamma band and prove that an ING mechanism in conjunction with interneuronal STDP might be a strong candidate for stable gamma oscillations in cortical and even hippocampal regions (Bragin et al., 1995). A future research endeavor would be the inclusion of fast firing excitatory pyramidal cells network along with our MCI model to confirm that the ING mechanism can indeed drive pyramidal cells to synchronize at gamma frequencies. Additionally, it would be of great interest to see if and how STDP can induce 1:1 and in-phase synchronization in non-MCI networks with more random architectures. In conclusion, this work provides further evidence that for significant heterogeneity in firing rates, STDP may be a viable alternative to other synchronization mechanisms such as gap junctions (Traub et al., 2001).

## Author contributions

ST, PK conceived the initial design of the work with additional input from SR. SR performed all the simulation experiments and analysis. SR wrote the manuscript with significant inputs from PK and ST.

## Funding

This research was funded by startup funds to ST from the Department of Pediatrics at the University of Florida; the intramural grant on Computational Biology at the University of Florida; and the Wilder Center of Excellence for Epilepsy Research and the Children's Miracle Network. PK was partially supported by the Eckis Professor Endowment at the University of Florida.

### Conflict of interest statement

The authors declare that the research was conducted in the absence of any commercial or financial relationships that could be construed as a potential conflict of interest.
